# Lighting from Top and Side Enhances Photosynthesis and Plant Performance by Improving Light Usage Efficiency

**DOI:** 10.3390/ijms23052448

**Published:** 2022-02-23

**Authors:** Jingli Yang, Jinnan Song, Byoung Ryong Jeong

**Affiliations:** 1 Department of Horticulture, Division of Applied Life Science (BK21 Four), Graduate School of Gyeongsang National University, Jinju 52828, Korea; yangmiaomiaode@gmail.com (J.Y.); jinnansong93@gmail.com (J.S.); 2 Institute of Agriculture and Life Science, Gyeongsang National University, Jinju 52828, Korea; 3 Research Institute of Life Science, Gyeongsang National University, Jinju 52828, Korea

**Keywords:** chrysanthemum, lighting direction, light usage efficiency, branching, flowering, photomorphogenesis, photosynthesis

## Abstract

Light is a critical environmental factor that influences plant growth and development, ranging from seed germination to flowering and fruiting. This study was carried out to explore how the optimal combination of various lighting directions increases the light usage efficiency and influences the plant morphophysiology, by investigating the plant growth parameters, leaf anatomy, epidermal morphology, stomatal properties, chlorophyll content, key physiological changes, and correlated gene expressions. In closed-type plant growth chambers, rooted cuttings of two chrysanthemum (*Chrysanthemum morifolium* Ramat.) cultivars, “Pearl Egg” and “Gaya Glory”, were subjected to a 10-h photoperiod with 600 μmol∙m^−2^·s^−1^ photosynthetic photon flux density (PPFD) provided by light-emitting diodes (LEDs) in each light-direction combination (top (1/1) (T), top (1/2) + side (1/2) (TS), top (1/2) + bottom (1/2) (TB), side (1/2) + bottom (1/2) (SB), and top (1/3) + side (1/3) + bottom (1/3) (TSB)). The TS lighting significantly enhanced the morphophysiological performance, compared to the other lighting direction combinations. Notably, the excellent branch formation and earlier flowering were induced by the TS lighting in both “Pearl Egg” and “Gaya Glory” plants.

## 1. Introduction

Since plants are sessile, external variables unavoidably have a significant impact on their physiology and development. Plants have evolved complex ways of sensing the environmental stimuli and converting them into internal signaling pathways in order to adapt to and survive in a changing environment. Light is one of the most important environmental cues, affecting practically every stage of a plant’s lifetime. After germination from the soil, etiolated growth causes the germinated seedling to grow toward the soil surface in search of light. When exposed to light, the seedling goes through photomorphogenesis, which includes de-etiolation, chlorophyll synthesis, and chloroplast growth, all of which help the seedling become an independent autotroph. Thus, light is used as a sophisticated signaling input to influence plant physiology and growth, in addition to being the only energy source for CO_2_ fixation during photosynthesis. Plant development and growth are influenced by both the quality and amount of the incident light. Furthermore, numerous photoreceptors are required for plants to appropriately process the light input [1,2]. For most plants, the leaves are where most photoreceptors exist and transform the energy. Leaves sense and capture the sun’s energy with the help of multiple photoreceptors and chlorophyll in the leaf cells, respectively. In a process known as photosynthesis, chlorophyll captures and absorbs the energy from the sun’s light. Leaves usually have a large surface to collect as much sunlight as possible [3].

**Figure 1:**
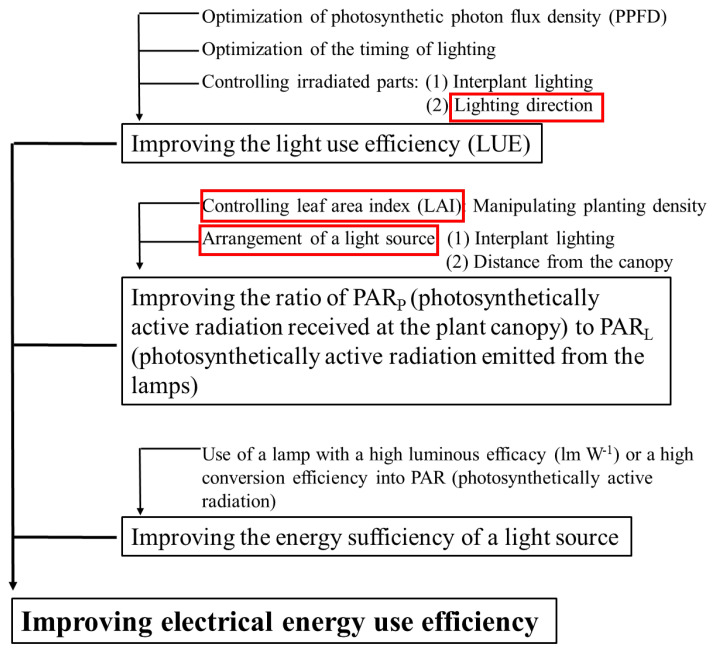
Factors affecting the electrical energy use efficiency.

A controlled environment with artificial lighting (CEAL) is an airtight facility for growing plants indoors under artificially regulated circumstances, such as plant factories and growth chambers [4,5,6]. These facilities are increasingly being utilized for commercial flowering plants, leafy vegetables, fruit production, plant science research, and high-quality transplant production [7,8,9,10]. CEAL is critical for maximizing the efficiency of resources utilized in production, particularly light usage efficiency. However, little attention has been paid to the efficiency of the lighting [11]. Using lighting with high light effectiveness is one possible approach. In addition, the placement of the light sources and/or the plant canopy structure being irradiated also affects the lighting efficiency [3]. Various methods can be considered to improve the electrical energy use efficiency (Figure 1) [12]. Consistent with our previous study, selecting the optimal lighting position, adjusting for a favorable lighting direction, and controlling the maximum leaf area exposed to light (the boxes marked in red Figure 1) enhanced plant performance via increased photosynthesis through a higher light use efficiency [13,14].

Variations in the light direction affect plant morphophysiology. The light interception is directly determined by leaf orientation. Phototropism-induced variations in the leaf angle and leaf movement (epinastic or hyponastic) have been proposed as a means of increasing the photosynthetic capacity, efficiency, and carbon gain in light competition conditions [15,16,17]. The epidermal cells in the midribs were stimulated as a result of the changes in the leaf angle, which further influenced the stomatal status. Stomatal density and size are thought to be indications of a plant’s acclimatization and adaptability to different conditions [18,19]. The optimal strategy for getting the highest stomatal conductance at low CO_2_ concentrations which is lucrative for high photosynthetic efficiency is to have a dense, open, and smaller stoma [20].

Furthermore, measures of chlorophyll fluorescence have been recognized as a useful and informative indicator of photosynthetic light response efficiency. Under prevailing light and shadow conditions, chlorophyll fluorescence is principally and effectively used to evaluate the potential quantum yield of photosystem II and photoinhibition [21]. Inappropriate lighting or shade has a significant influence on the function and structure of the photosynthetic apparatus [22], reduces chlorophyll fluorescence by lowering the thicknesses of leaves, palisades, and spongy tissues, and inhibits the energy transfer from PSII to PSI [22,23].

Moreover, plants have evolved complex acclimatization mechanisms to address adverse environments, such as the reactive oxygen species (ROS)-scavenging enzymatic antioxidant system [24]. Under stressful situations, the equilibrium between ROS formation and the antioxidants’ quenching action is disrupted, resulting in oxidative damage [25]. Stronger antioxidant enzyme activity is usually associated with a greater capacity to remove ROS. Moreover, chlorophyll a is more sensitive to ROS than chlorophyll b is under stress conditions. ROS induces a direct decrease in the chlorophyll a and total chlorophyll concentration, affecting photosynthesis [26,27]. In addition, the amount and activity of major enzymes involved in CO_2_ fixation and RuBisCO-1, 5-bisphosphate (RuBP) regeneration, as well as the content and activity of light-capturing components, electron transport fragments, and energy transferring enzymes were used to assess the metabolic capability of photosynthesis in plants under diverse conditions [28,29,30,31,32,33]. RuBisCO (RuBP carboxylase or oxygenase) catalyzes the CO_2_ fixation in photosynthesis [34], which is directly engaged in the first phase of the Calvin Benson cycle and accounts for 12–35% of the leaf proteins, notably in C_3_ crop plants [35]. Previous studies have indicated a reduction in the RuBisCO amount or activity as the key biochemical limitation implicated in the shade-associated down-regulation of the net photosynthetic rate [28].

Numerous studies have confirmed that the light intensity, quality, duration, and source type, such as the sun or artificial lighting systems, affect plant growth and development [36,37,38]. However, studies have rarely focused on the influences of changes in the light usage efficiency induced by variations in the lighting direction combination on plant morphophysiology. According to previous studies, lettuces and chrysanthemums adapted to the solo light-direction variation from cellular to the individual level [13,14,39]. In this experiment, we investigated how chrysanthemum responds to the various lighting direction combinations to help fine-tune the growth environment for their development. Our study refers to the profound effects on the plant morphology, leaf internal structures, cellular characteristics, chlorophyll content, photosynthetic and chlorophyll fluoresces parameters, physiological changes, and transcriptional analysis of some targeted genes to investigate the optimum lighting direction combination for the growth and development of chrysanthemums. We will better understand the improvement of the light usage efficiency by studying the optimal lighting direction combination.

## 2. Results

### 2.1. Morphology and Plant Growth Parameters

**Figure 2:**
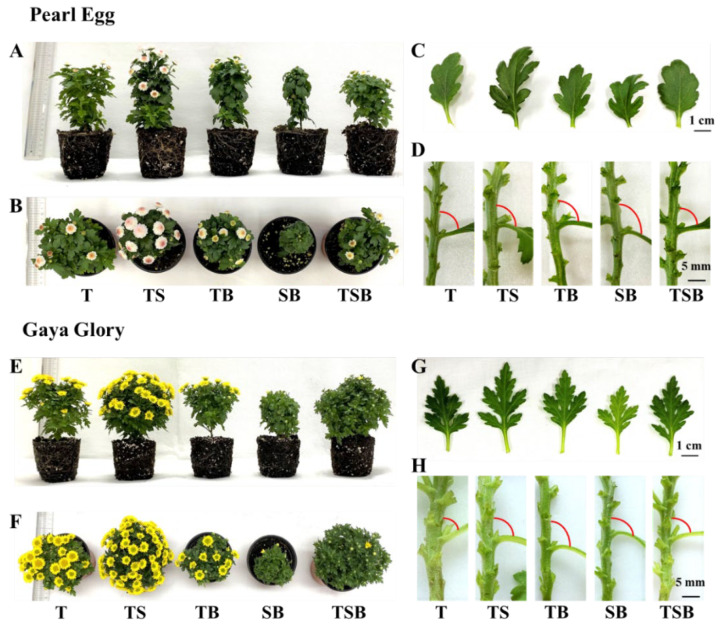
Changes in the phenotype and plant traits of chrysanthemum “Pearl Egg” and “Gaya Glory” plants as affected by the different lighting direction combinations. The plant morphology (**A**,**B**,**E**,**F**), leaf phenotype (**C**,**G**), and adaxial leaf petiole angle (**D**,**H**) of “Pearl Egg” and “Gaya Glory” as affected by the different lighting direction combinations after 45 days of cultivation. T, TS, TB, SB, and TSB, refer to the top (1/1), top (1/2) + side (1/2), top (1/2) + bottom (1/2), side (1/2) + bottom (1/2), and top (1/3) + side (1/3) + bottom (1/3) lighting, respectively. Bars indicate 1 cm or 5 mm.

The chrysanthemum plants were kept in the growth chamber for 45 days. The morphology and plant growth measurements of “Pearl Egg” and “Gaya Glory” plants at the end of the experiment are shown in Figure 2 and Table 1. The morphological characteristics of chrysanthemums were affected by the different lighting direction combinations (Figure 2). In the present experiment, the growth attributes of “Pearl Egg” and “Gaya Glory”, including the plant shoot, leaf, flower, and root-related parameters were significantly affected by the different lighting direction combinations (Table 1). Both chrysanthemums “Pearl Egg” and “Gaya Glory” produced flowers with all lighting direction combinations. However, there were substantial differences in the number of flowers in response to the different lighting direction combinations, especially between TS and SB. Relative to other lighting direction combinations, the TS resulted in the greatest number of flowers, branches, and leaves in both “Pearl Egg” and “Gaya Glory” (Figure 2A,B,E,F, and Table 1). Specifically, the largest length and width of leaves, and also the stem diameter were observed in response to the TS lighting (Figure 2C,D,G,H, and Table 1). Moreover, the TS lighting considerably promoted early flowering and markedly enhanced plant growth, evidenced by increased shoot height, plant biomass, root development, etc., regardless of the cultivar (Table 1). The TS and SB lighting led to the most significant differences in the morphology and development of chrysanthemums. The chrysanthemum morphology and development were also influenced by the other lighting direction combinations; particularly, the TSB lighting delayed flowering in both “Pearl Egg” and “Gaya Glory”. In addition, the plant leaves bent toward the light due to the phototaxis, which resulted in the adaxial leaf petiole angle being considerably increased with the T, TSB, TS, TB, and SB lighting. The epinastic development of chrysanthemum leaves was caused by the changed lighting direction combinations, which adjusted the adaxial leaf petiole angle to increase the light absorption area of chrysanthemum leaves, which further promoted plant growth and enhanced the photosynthetic activity in chrysanthemums. Altogether, the combinations of various lighting directions observably affected the morphology and growth of plants, and the optimum combination positively promoted chrysanthemum development.

### 2.2. Leaf Anatomy

**Figure 3:**
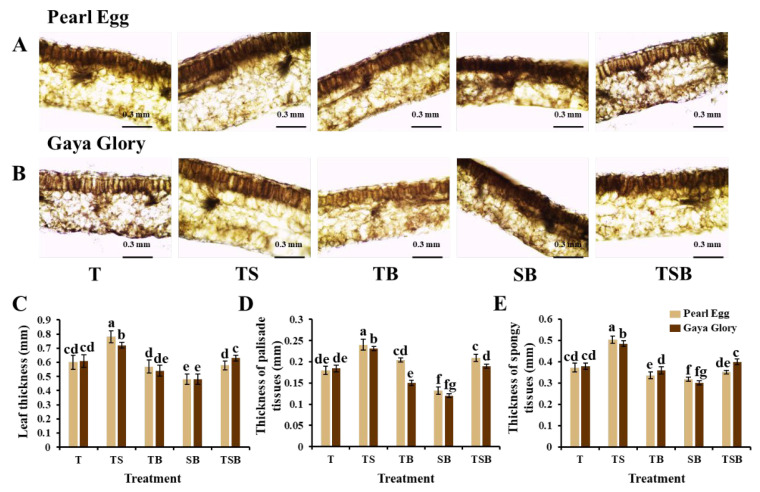
Changes in the leaf structure of chrysanthemum “Pearl Egg” and “Gaya Glory” plants as affected by the different lighting direction combinations. The leaf structure (**A**,**B**); the thickness of leaves, palisade tissues, and spongy tissues (**C**–**E**). T, TS, TB, SB, and TSB, refer to top (1/1), top (1/2) + side (1/2), top (1/2) + bottom (1/2), side (1/2) + bottom (1/2), and top (1/3) + side (1/3) + bottom (1/3) lighting, respectively. Vertical bars indicate the means ± standard error (*n* = 6). Different lowercase letters indicate significant separation within treatments by Duncan’s multiple range test at *p* ≤ 0.05. Bars indicate 0.3 mm.

The light quality, quantity, intensity, and especially light direction noticeably affect leaf anatomy. In this study, significant differences in the thicknesses of leaves, palisade tissues, and spongy tissues were observed in response to all five lighting direction combinations in both “Pearl Egg” and “Gaya Glory” plants (Figure 3A–E). The TS lighting observably increased leaf thickness and improved the development of palisade and spongy tissues. When compared to the SB lighting, TS light resulted in well-developed leaves with clear and compact structures. Moreover, leaf structures were also affected by the other three lighting direction combinations, and there were moderate differences in the leaf anatomy in response to the T, TB, and TSB lighting. These findings indicated that non-optimal lighting direction combinations negatively affected the chrysanthemum leaf tissue size, while an optimum combination positively enhanced the leaf tissue structures.

### 2.3. Morphological Characteristics of the Epidermal Cells and Stomata

**Figure 4:**
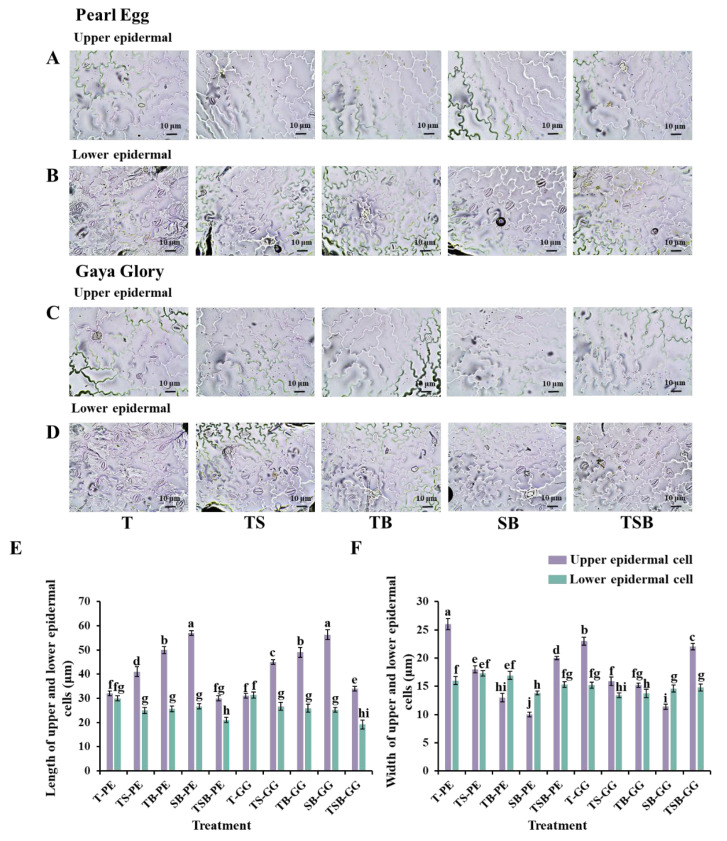
Changes in the epidermal cell morphology of chrysanthemum “Pearl Egg” and “Gaya Glory” plants as affected by the different lighting direction combinations. Micrographs of upper and lower epidermal cells of “Pearl Egg” (**A**,**B**) and “Gaya Glory” (**C**,**D**), length of upper and lower epidermal cells (**E**), and width of upper and lower epidermal cells (**F**) of “Pearl Egg” and “Gaya Glory” as affected by the different lighting direction combinations after 45 days of cultivation. T, TS, TB, SB, and TSB-PE/GG, refer to the top (1/1), top (1/2) + side (1/2), top (1/2) + bottom (1/2), side (1/2) + bottom (1/2), and top (1/3) + side (1/3) + bottom (1/3) lighting, respectively. Vertical bars indicate the means ± standard error (*n* = 6). Different lowercase letters indicate significant separations within treatments by Duncan’s multiple range test at *p* ≤ 0.05. Bars indicate 10 μm.

Due to the positive phototaxis, the aboveground parts of plants will grow toward the light. Leaves grow downward to receive more light when the light comes from the side and bottom, which further influences the morphology of the epidermis in the leaf midribs (Figure 4A–F). In the present study, in both “Pearl Egg” and “Gaya Glory” plants, there was a positive relationship between the adaxial leaf petiole angle and the upper epidermal cell length. Except for the T lighting, the other lighting direction combinations, especially the SB and TB lighting, resulted in larger adaxial leaf petiole angles, and increased upper epidermal cell elongation is a response to light conditions. Meanwhile, the lower epidermis was squeezed by blade downward curl, inducing wide and short cells. Overall, the different lighting direction combinations greatly affected the morphology of the upper epidermis, in contrast to the relatively milder or insignificant changes in the lower epidermis in response to the different lighting direction combinations, regardless of the cultivar.

**Figure 5:**
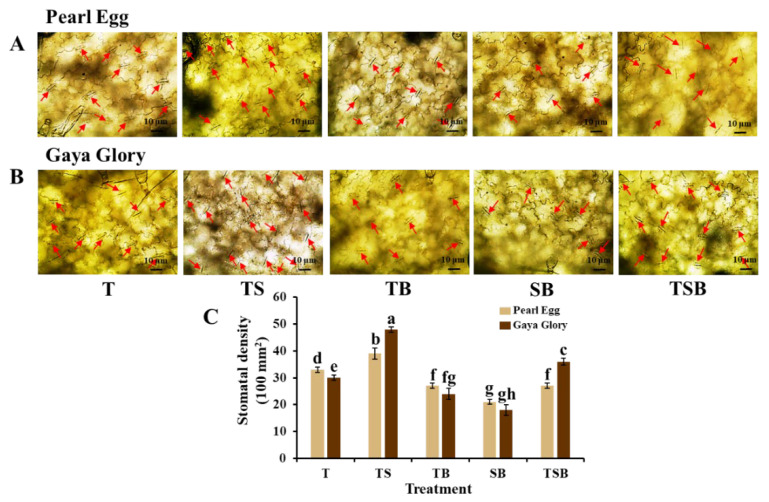
Changes in the stomatal density of chrysanthemum ‘Pearl Egg’ and ‘Gaya Glory’ plants as affected by the different lighting direction combinations. Micrographs of stomata (20 ×) (**A**,**B**) and stomatal density (**C**) of “Pearl Egg” and “Gaya Glory” as affected by the different lighting direction combinations after 45 days of cultivation. The red arrows indicate the stomata. T, TS, TB, SB, and TSB, refer top (1/1), top (1/2) + side (1/2), top (1/2) + bottom (1/2), side (1/2) + bottom (1/2), and top (1/3) + side (1/3) + bottom (1/3) lighting, respectively. Vertical bars indicate the means ± standard error (*n* = 6). Different lowercase letters indicate significant separations within treatments by Duncan’s multiple range test at *p* ≤ 0.05. Bars indicate 10 μm.

**Figure 6:**
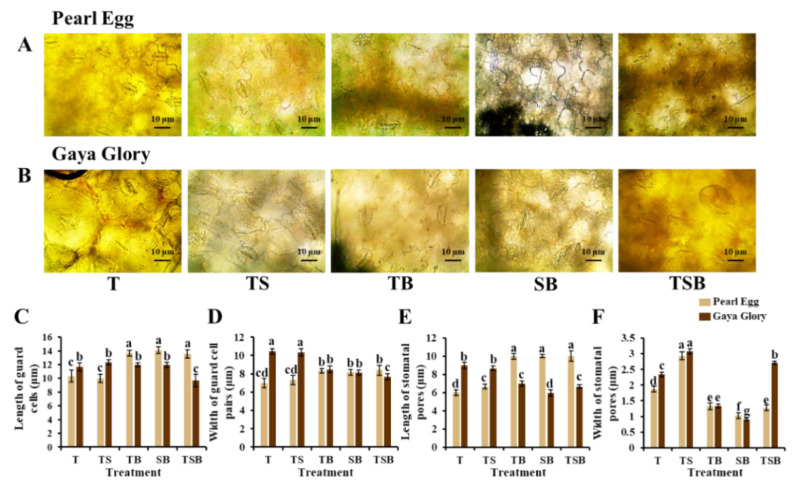
Changes in the stomatal morphology and parameters of chrysanthemum “Pearl Egg” and “Gaya Glory” plants as affected the different lighting direction combinations. Micrographs of stomatal morphology (40×) (**A**,**B**), length of guard cells (**C**), width of guard cell pairs (**D**), length of stomatal pores (**E**), and width of stomatal pores (**F**) of “Pearl Egg” and “Gaya Glory” as affected by the different lighting direction combinations after 45 days of cultivation. T, TS, TB, SB, and TSB, refer top (1/1), top (1/2) + side (1/2), top (1/2) + bottom (1/2), side (1/2) + bottom (1/2), and top (1/3) + side (1/3) + bottom (1/3) lighting, respectively. Vertical bars indicate the means ± standard error (*n* = 6). Different lowercase letters indicate significant separations within treatments by Duncan’s multiple range test at *p* ≤ 0.05. Bars indicate 10 μm.

In addition, to further explore the epidermal cellular morphology response to various lighting direction combinations, we examined the stomatal characteristics in both “Pearl Egg” and “Gaya Glory” plants as affected by the different lighting direction combinations after 45 days (Figure 5 and Figure 6). Compared to other combinations, especially the SB and TB lighting, the TS lighting observably increased the stomatal density. The stomatal density in response to the T and TSB lighting changed smoothly (Figure 5). Moreover, the TS lighting markedly promoted stomata opening in both chrysanthemum cultivars (Figure 6). Well-opened stomata were also observed in response to the TSB and T lighting in “Gaya Glory” plants but not in “Pearl Egg”. Furthermore, there were irregular changes in the stomatal size in response to various lighting direction combinations in both “Pearl Egg” and “Gaya Glory” plants. It was demonstrated that the extent of the influences of various lighting direction combinations on stomatal properties was strongly dependent on the cultivar. Altogether, the TS lighting positively affected the stomatal density and opening, which are vital factors in positively enhancing the photosynthetic efficiency.

### 2.4. Chlorophyll Content

**Figure 7:**
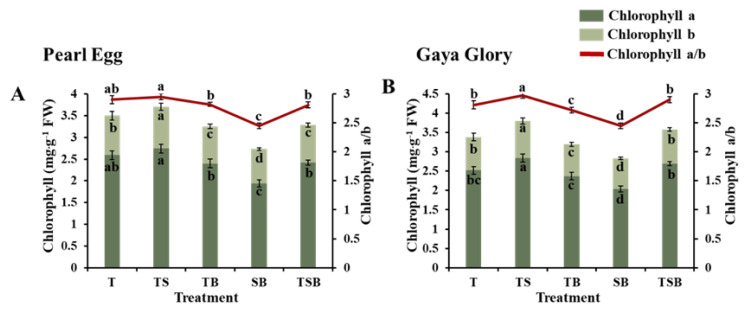
Changes in the chlorophyll content of chrysanthemum “Pearl Egg” (**A**) and “Gaya Glory” (**B**) plants in response to the different lighting direction combinations after 45 days of cultivation. T, TS, TB, SB, and TSB, refer top (1/1), top (1/2) + side (1/2), top (1/2) + bottom (1/2), side (1/2) + bottom (1/2), and top (1/3) + side (1/3) + bottom (1/3) lighting, respectively. Vertical bars indicate the means ± standard error (*n* = 6). Different lowercase letters indicate the significant separation within treatments by Duncan’s multiple range test at *p* ≤ 0.05.

The chlorophyll (Chl) content is another important factor related to photosynthesis. The Chl content in chrysanthemum leaves was considerably affected by the different lighting direction combinations (Figure 7). In this experiment, changing the lighting from SB to TS increased the Chl a, Chl b, Chl a + b contents, and the Chl a/b ratio. In addition, the Chl a, b, and a + b contents in chrysanthemum leaves were found to be insignificantly affected by the T, TB, and TSB lighting, but the Chl a, b, a + b contents, and Chl a/b were always higher in response to the TS lighting than to the SB lighting. These improvements suggest a direct relationship of the chlorophyll content in both “Pearl Egg” and “Gaya Glory” plants with the changes in the lighting direction combinations.

### 2.5. Photosynthetic and Chlorophyll Fluorescence Characteristics

Table 2 shows the photosynthetic characteristics of chrysanthemums in response to the various lighting direction combinations. The maximum and minimum values of *P*_n_, *T*_r_, *G*_s_, and *C*_i_ in both “Pearl Egg” and “Gaya Glory” plants appeared in response to the TS and SB lighting, respectively. There were irregular differences in the *P*_n_, *T*_r_, *G*_s_, and *C*_i_ values in response to the T, TB, and TSB lighting in both chrysanthemums, but these parameters were always higher than those in response to the SB lighting and lower than those in response to the TS lighting. This increase in the net photosynthetic rate indicates that TS lighting is highly effective for photosynthesis. These changes may be due to the enhancement of the stomatal properties and chlorophyll contents by the TS lighting.

The absorbed radiation energy in both “Pearl Egg” and “Gaya Glory” plants was studied in response to the various light direction combinations (Table 3). In this experiment, the chlorophyll fluorescence parameters *F*_v_/*F*_m_, *F*_v_’/*F*_m_’, NPQ, and *qP* were notably changed in response to the different lighting direction combinations. Independent of the cultivar, the *F*_v_/*F*_m_, *F*_v_’/*F*_m_’, NPQ, and *qP* of chrysanthemum leaves were considerably higher in response to the TS lighting than in response to the SB lighting. These values were insignificantly different in response to the T, TB, and TSB lighting. Moreover, these parameters were not very different between “Pearl Egg” and “Gaya Glory” when they responded to the T, TB, and TSB lighting. For “Pearl Egg”, the values of *F*_v_/*F*_m_, *F*_v_’/*F*_m_’, NPQ, and *qP* were increased when the lighting changed from TB or TSB to T, while these values were enhanced when T or TB changed to TSB lighting in “Gaya Glory” plants, but these were always lower than those observed in response to the TS lighting. Overall, our results show that an appropriate lighting direction combination is critical for increasing the chlorophyll fluorescence characteristics and photosynthetic capacity in chrysanthemum plants.

### 2.6. Carbohydrates and Soluble Proteins

**Figure 8:**
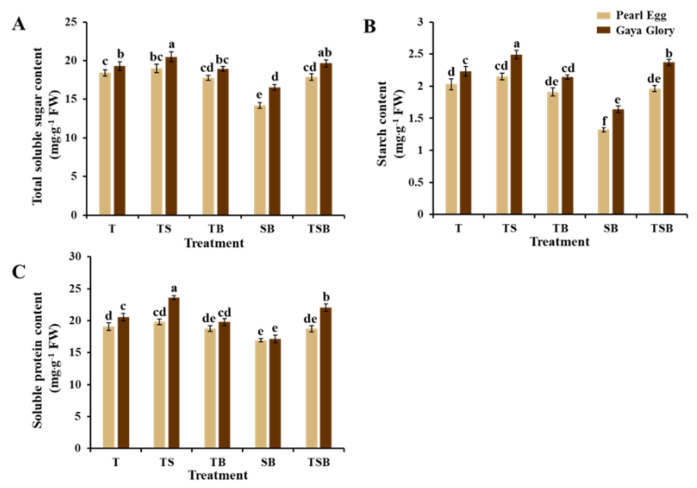
Changes in the content of carbohydrates (**A**,**B**) and soluble proteins (**C**) of chrysanthemum “Pearl Egg” and “Gaya Glory” plants as affected by the different lighting direction combinations after 45 days of cultivation. T, TS, TB, SB, and TSB, refer to top (1/1), top (1/2) + side (1/2), top (1/2) + bottom (1/2), side (1/2) + bottom (1/2), and top (1/3) + side (1/3) + bottom (1/3) lighting, respectively. Vertical bars indicate the means ± standard error (*n* = 6). Different lowercase letters indicate significant separations within treatments by Duncan’s multiple range test at *p* ≤ 0.05.

From the previous results, it can be seen that the various lighting direction combinations induced differences in the photosynthetic efficiency (Table 2 and Table 3). We determined the contents of carbohydrates and soluble proteins in both “Pearl Egg” and “Gaya Glory” plant leaves to further explore the effects of the lighting direction combination on photosynthesis in chrysanthemum (Figure 8). In our study, as expected, the lighting direction combinations affected the accumulation of carbohydrates and soluble proteins in both cultivars. For “Pearl Egg”, the total soluble sugar and starch contents were significantly increased when the lighting changed from SB, TSB, and TB to T, and especially TS. The maximum and minimum values of carbohydrates were respectively observed in response to TS and SB lighting, and there were insignificant differences in the total soluble sugar and starch contents in response to TB and TSB lighting. Moreover, the soluble protein content in “Pearl Egg” displayed a similar tendency as that of carbohydrate contents. For “Gaya Glory”, the greatest values of soluble sugar, starch, and soluble proteins were observed in response to the TS lighting, and the lowest values were observed in response to the SB lighting. In addition, except for the TS and SB lighting, the contents of carbohydrates and soluble proteins increased from the TB, T, to TSB lighting. Overall, this trend of increased carbohydrates and soluble proteins proved the epinastic movement of chrysanthemum leaves because as previously mentioned, the different lighting direction combinations adjusted the adaxial leaf petiole angle and increased the light absorption area of chrysanthemum leaves which in turn increased the content of carbohydrates and soluble proteins due to the higher photosynthetic activity in chrysanthemum plants.

### 2.7. Enzymatic Activity

**Figure 9:**
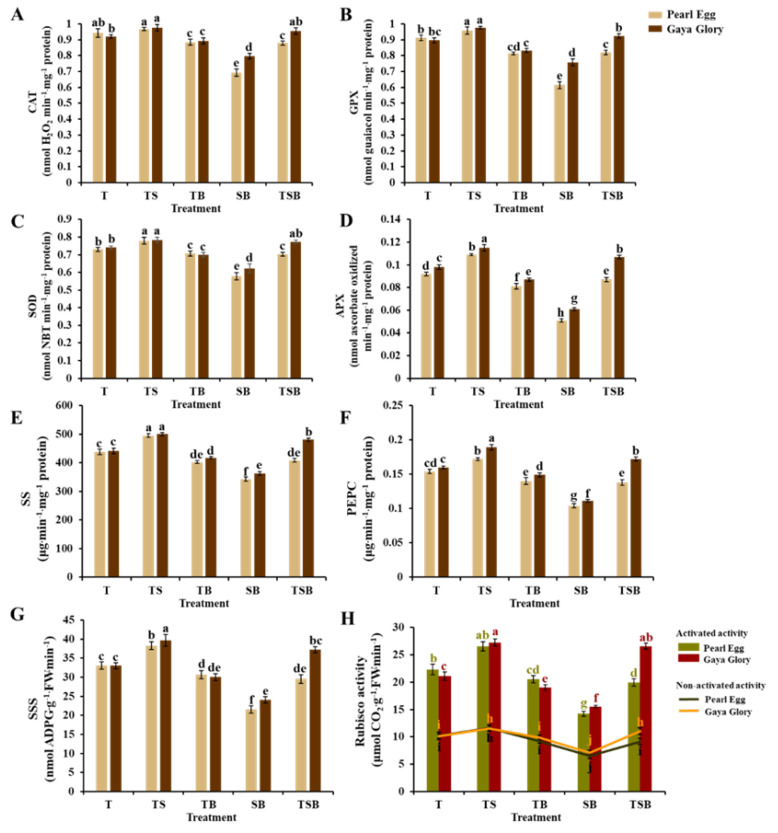
Changes in the enzymatic activities in chrysanthemum “Pearl Egg” and “Gaya Glory” as affected by the different lighting direction combinations after 45 days of cultivation. The ROS scavenging enzymatic activities: Catalase (CAT) (**A**), guaiacol peroxidase (GPX) (**B**), superoxide peroxidase (SOD) (**C**), and ascorbate peroxidase (APX) (**D**). Sucrose synthesis enzymatic activities: Sucrose synthase (SS) (**E**) and phosphoenolpyruvate carboxykinase (PEPC) (**F**). Starch synthesis enzymatic activities: Soluble starch synthase (SSS) (**G**). Photosynthesis enzymatic activities: Activated and non-activated activity of RuBisCO (**H**). T, TS, TB, SB, and TSB, refer top (1/1), top (1/2) + side (1/2), top (1/2) + bottom (1/2), side (1/2) + bottom (1/2), and top (1/3) + side (1/3) + bottom (1/3) lighting, respectively. Vertical bars indicate the means ± standard error (*n* = 6). Different lowercase letters indicate significant separations within treatments by Duncan’s multiple range test at *p* ≤ 0.05.

In this experiment, significant differences in the ROS scavenging enzymatic activities (catalase (CAT), guaiacol peroxidase (GPX), super-oxide peroxidase (SOD), and ascorbate peroxidase (APX)), sucrose synthesis enzymatic activities (sucrose synthase (SS) and phosphoenolpyruvate carboxykinase (PEPC)), starch synthesis enzymatic activities (soluble starch synthase (SSS)), and photosynthesis enzymatic activities (activated and non-activated activity of RuBisCO) were observed in response to the various lighting direction combinations (Figure 9). The CAT, GPX, SOD, and APX activities of both “Pearl Egg” and “Gaya Glory” plants gradually increased as the lighting changed from SB to TS, and higher values were observed in response to the TS lighting than in response to the other lighting direction combinations (Figure 9A–D). For “Pearl Egg” plants, the enzymatic activities were higher in response to the T lighting than in response to the TB or TSB lighting. Moreover, for “Gaya Glory”, the enzymatic activities were higher in response to the TSB lighting than in response to the T and TB lighting. In addition, the increased tendency of SS, PEPC, SSS, and RuBisCO activities was similar to that of ROS scavenging enzymatic activities in “Pearl Egg” and “Gaya Glory” (Figure 9E–H). Enhancement in those enzymatic activities occurred in response to all lighting direction combinations, and the acceleration was greater in response to the TS lighting than to the T, TB, TSB, and especially SB lighting. These findings imply that the enzymatic activities were directly linked to variations in the lighting direction combinations. The TS lighting may be more effective in stimulating the enzymatic activities of chrysanthemums.

### 2.8. Gene Expression

**Figure 10:**
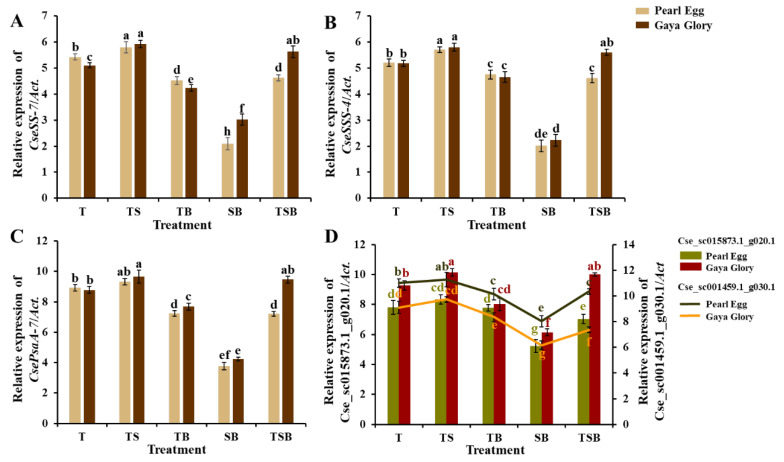
Changes in the gene expression levels in chrysanthemum “Pearl Egg” and “Gaya Glory” as affected by the different lighting direction combinations after 45 days of cultivation. Sucrose synthesis related genes: *CseSS-7* (**A**). Starch synthesis related genes: *CseSSS-4* (**B**). Photosynthesis-related genes: *CsePsaA-7* (**C**). Flowering related genes: *Cse_sc015873.1_g020.1* and *Cse_sc001459.1_g030.1* (**D**). T, TS, TB, SB, and TSB, refer to the top (1/1), top (1/2) + side (1/2), top (1/2) + bottom (1/2), side (1/2) + bottom (1/2), and top (1/3) + side (1/3) + bottom (1/3) lighting, respectively. Vertical bars indicate the means ± standard error (*n* = 6). Different lowercase letters indicate significant separations within treatments by Duncan’s multiple range test at *p* ≤ 0.05.

The expression levels of one gene related to sucrose synthesis (*CseSS-7*), one gene related to starch synthesis (*CseSSS-4*), one gene related to photosynthesis (*CsePsaA-7*), and two genes related to flowering (*Cse_sc015873.1_g020.1* and *Cse_sc001459.1_g030.1*) were investigated in our study. The relative expression levels of all 5 genes for sucrose synthesis, starch synthesis, photosynthesis, and flowering were up-regulated as the lighting direction changed from SB to TS in both “Pearl Egg” and “Gaya Glory” (Figure 10). For “Pearl Egg” plants, these gene expression levels were higher in response to the T lighting than in response to the TB and TSB lighting. Moreover, for “Gaya Glory”, these gene expression levels were higher in response to the TSB lighting than in response to the T and TB lighting. The tendencies of the gene expression levels in response to the different lighting direction combinations were consistent with the corresponding enzymatic activities.

## 3. Discussion

### 3.1. Variations in the Lighting Direction Combinations: Their Effects on Morphology and Growth Parameters of Chrysanthemum “Pearl Egg” and “Gaya Glory”

In the current research, the “Pearl Egg” and “Gaya Glory” plants exhibited a generally consistent tendency in response to the various lighting direction combinations. Most notably, the TS lighting significantly promoted earlier flowering and induced an abundance of flowers in chrysanthemums (Figure 2A,B,E,F, and Table 1). The photoperiodic pathway, vernalization pathway, temperature pathway, autonomous pathway, gibberellin pathway, and age pathway have all been found in plants as flowering regulating processes. Light signals in leaves are detected by phytochromes, cryptochromes, ZTL/FKF1/LKP2, etc., which are then transmitted to the circadian clock. The photoreceptors eventually regulate flowering directly or indirectly after signals integrate across various flowering pathways [40,41,42,43]. There are multiple photoreceptors that can respond to various light wavelengths, located on the upper leaf surface. The resultant regulators are subsequently transferred from the phloem to the apical meristem, where they combine with a series of proteins to produce a transcriptionally active flowering complex that drives flowering [44]. The chrysanthemums grown with the TS lighting induced the greatest number of leaves with a larger size that can efficiently capture and use the available light, and promote the expression of flowering-related genes. This may explain why the TS lighting exerted such a strong positive influence on flowering. 

Interestingly, not only the flowering but also the branching positively responded to the TS lighting. Compared to the other four lighting direction combinations, the TS lighting particularly enhanced branching (Table 1). As was observed in our previous studies, adjusting the lighting direction from bottom and top to the side increased the light use efficiency, which significantly promoted flowering, branching, runner formation, and plant performance in chrysanthemum and strawberry [13,14]. In the present experiment, the chrysanthemums grown with the T lighting received light only from the top, which well-developed the terminal bud of the stem and made it grow preferentially, while the auxin produced was polar-transported to the lateral bud while inhibiting the lateral bud production, which is also known as apical dominance [45]. This may be the reason why the T lighting induced fewer branches than the TS lighting did. Moreover, the TS lighting resulted in the greatest light efficiency, which further induced well-developed terminal buds and lateral buds, causing higher bio-activities and promoting phytohormone cycles. Meanwhile, the well-developed lateral shoot preferentially receives branch-promoted hormones such as cytokinins transported from the root, thus promoting the quantity and quality of the lateral buds [46,47,48]. In addition, according to the nutrition theory that K. Gerber proposed, the well-developed bud cells rapidly grow, vigorously metabolize, and require more nutrients. In our experiment, chrysanthemums grown with the TS lighting showed the greatest bud formation, which indicates that these buds received sufficient light, metabolized vigorously, rapidly grew the cells, and therefore preferentially received more nutrients, and their growth was further facilitated. Taken together, adjusting the contact surface between the leaf upper surface and light is an important factor to efficiently utilize light [12]. The TS lighting improved the lateral bud induction and substantially increased the number of branches and leaves by providing more favorable conditions.

Furthermore, the TS lighting remarkably enhanced the shoot fresh and dry weights but decreased the stem internode length (Table 1), which is like results of earlier research where the sideward lighting induced considerably shorter stems but increased the dry weight of in vitro micro-propagated potato plantlets when compared to those grown with top lighting [49]. In addition, the greatest stem diameter and well-developed roots of chrysanthemums observed in response to the TS lighting may be up-regulated by higher photosynthesis, which supplies adequate energy to the stems and roots [50], combining endogenous plant hormones and complicated molecular regulatory networks [51,52]. Overall, these results showed that the TS lighting greatly improved the chrysanthemum morphology, promoting branching and flowering, and is still important for enhancing plant performance.

### 3.2. Variations in the Lighting Direction Combinations: Their Effects on the Leaf Anatomy, Epidermis, Stoma, and Chlorophyll Content of Chrysanthemum “Pearl Egg” and “Gaya Glory”

Plants’ morphological structures and physiological functions are well known to be related. The morphological properties of photosynthetic structures in leaves are intimately connected to the variations in the photosynthetic rate. The most well-developed leaf structures were obtained in both “Pearl Egg” and “Gaya Glory” plants in response to the TS lighting (Figure 3). Moreover, the TS lighting most significantly increased the thicknesses of leaves, spongy tissues, and palisade tissues. The high light interception capacity and photosynthetic efficiency are ensured by the greater leaf area. The photosynthetic rate was affected by the leaf area and the quantity of carbon partitioned to thicker leaves, which further aided in the development of the leaf structures [53,54]. Those enhancements in the thicknesses of leaves and spongy tissues in response to the TS lighting may be connected to the well-developed mesophyll tissues [55]. The SB lighting resulted in leaves with smaller cell sizes and loose cell layers, resulting in low palisade and spongy tissue thicknesses, perhaps due to lower cell growth and reduced cell layer number in the mesophyll tissues [56]. Consequently, the TS lighting significantly strengthened the leaf structures, which helped improve the photosynthetic capacity in chrysanthemums.

The TS lighting improved the elongation of the palisade and spongy tissues which enhanced the attachment region of chloroplasts, thereby increasing the chlorophyll content in chrysanthemum leaves [57]. Chlorophyll is one of the most important factors related to photosynthesis. Notable improvements were observed in the Chl a, b, and a + b contents in response to the TS lighting (Figure 7), which were closely associated with the development of the leaf structures. These findings are consistent with those reported in other research [58,59].

Morphology of the epidermis will be affected by variations in the lighting direction [13,14,39]. Plant shoots, especially the leaves, bend toward the light to be able to capture and more efficiently use the available light due to the positive phototropism [60,61]. When chrysanthemums receive light coming from different directions, particularly the oblique directions, the adaxial leaf petiole angle changes and bends the leaves toward the light source (Figure 2D,H). The prolate upper epidermal cells in midribs were observed to be stimulated, while the lower epidermis was squeezed, inducing wide, flat cells (Figure 4).

Stomata are gas exchange pores in the epidermis of leaves, stems, and other organs, which is correlated with photosynthesis. In our study, the leaf movement led to changes in the leaf angle due to phototropism, and further stimulated the epidermal cells and stomatal state. The chrysanthemums grown with the TS lighting exhibited the greatest stomatal density with open pores (Figure 5 and Figure 6). Consequently, because of the excellent stomatal characteristics, higher photosynthetic efficiency and strong stress resistance can be observed in chrysanthemums grown with the TS lighting [20,62,63]. Thereby, the greatest plant performance was observed in response to the TS lighting (Table 1).

Altogether, the differences in the leaf anatomy, epidermis, stoma, and chlorophyll content in response to variations in the lighting direction suggest that the structural components of leaves are the main targets of light by adjusting the leaf anatomy. Chrysanthemums performed the best in response to the TS lighting.

### 3.3. Variations in the Lighting Direction Combinations: Their Effects on Photosynthesis and Primary Metabolite Yields of Chrysanthemum “Pearl Egg” and “Gaya Glory”

In this study, the TS lighting substantially enhanced the *P*_n_, *T*_r_, *G*_s_, and *C*_i_ levels in chrysanthemums (Table 2). Improved photosynthetic characteristics led to increased carbon gain and chrysanthemum growth [64]. Moreover, the well-developed leaf structures, abundant chlorophyll content, and higher density of opened stomata were closely associated with the enhancement of the net photosynthetic rate in chrysanthemums in response to the TS lighting [65,66,67].

A greater number of electrons flowing through PSII is always associated with an increased photosynthetic capability [37]. Because of its sensitivity and convenience, chlorophyll fluorescence properties are the most important component in photosynthetic regulation and plant responses to environmental variables [68]. The fluorescence characteristics of chlorophyll are intimately connected to many photosynthetic processes, and the effects of any stress on a specific photosynthesis process may be represented by the fluorescence kinetics of chlorophyllin [69]. Previous research has found a considerable positive linear association between the fluorescence characteristics and chlorophyll concentration in living plant leaves [70]. Similar results were obtained in our research, where the improvements of chlorophyll fluorescence characteristics were observed in both “Pearl Egg” and “Gaya Glory” plants in response to the TS lighting (Table 3). These outcomes uncover that an optimum lighting direction combination upgrades the proficiency of PSII and accordingly could further develop photosynthesis by advancing the energy transport from PSII to PSI.

Furthermore, in both “Pearl Egg” and “Gaya Glory” plants, the variations in the lighting direction combination affected the accumulation of the primary metabolites. Carbohydrates, such as starch and soluble sugars, are an immediate aftereffect of efficient photosynthesis, and carbohydrate amassing is crucial for plant growth, development, and morphology [71]. The soluble protein content is a significant physiological and biochemical metric, as well as an important indication for understanding the overall plant metabolism. The TS lighting increased the carbohydrate and soluble protein levels (Figure 8), which was a result of a combination of splendid stomatal characteristics, excellent chlorophyll concentrations, and prominent light usage efficiency. These conclusions were in general agreement with those in prior studies, that the optimal lighting direction will improve the accumulation of the primary metabolites [13,14].

### 3.4. Variations in the Lighting Direction Combinations: Their Effects on Enzymatic Activities and Gene Expressions in Chrysanthemum “Pearl Egg” and “Gaya Glory”

Plants have developed complex acclimatization mechanisms to resist adverse environments, such as the ROS (reactive oxygen species) scavenged enzymatic antioxidant system [24]. ROS creation is a typical peculiarity in plants under stresses. The balance between ROS creation and quenching activities of antioxidants is disturbed, thus resulting in oxidative damages when plants are under such adverse circumstances [25]. Higher antioxidant enzyme activities are usually associated with a stronger capacity to remove ROS.

Moreover, Chl a is more vulnerable to ROS than Chl b, and ROS induced direct degradation of Chl a and total chlorophyll concentrations under stress circumstances [26,27]. In our experiment, a highly active ROS scavenging system involving CAT, GPX, SOD, and APX, occurred under the TS lighting (Figure 9A–D). According to a comprehensive analysis of the previous results, the TS lighting effectively improved the chlorophyll content, the antioxidant capacity of the antioxidant enzyme system, and the resistance to stresses in chrysanthemums, which were consistent with the positive correlation between the chlorophyll content and the activity of the ROS scavenging antioxidant system.

In addition, results from this study demonstrated that the enzymatic activities of key enzymes related to sucrose synthesis (SS and PEPC), starch synthesis (SSS), and photosynthesis (RuBisCO) processes significantly changed with the variations in the lighting direction combinations. Higher activity levels of these enzymes were observed in response to the TS lighting (Figure 9E–H). These results are in line with the findings of previous reports where changes in the lighting direction equally played major roles in accelerating the activities of SS, PEPC, SSS, and RuBisCO [13,14]. Thus, the activities of SS, PEPC, and SSS may impact plant biomass and the net photosynthetic rate, which were mostly influenced by the TS lighting, and controlled cell elongation and division in plants by controlling the expression of numerous genes. These findings suggest that the activity of those enzymes, in conjunction with other plant responses to the TS lighting increased the carbohydrate content. Furthermore, the TS lighting enhanced the activity of RuBisCO in this study (Figure 9H). The increased RuBisCO activity in chrysanthemum plants in response to the TS lighting demonstrated that the higher net photosynthetic rate is directly connected to the RuBisCO activity in changing conditions [72]. Chrysanthemums grown under the TS lighting can be considered more effective at enzymatic activities in our experiment.

The relative gene expression levels of *CseSS-7* and *CseSSS-4*, respectively involved in sucrose and starch synthesis were up-regulated by the TS lighting in both “Pearl Egg” and “Gaya Glory” plants (Figure 10A,B). Perhaps there is a direct relation between the carbohydrate-synthesis enzymatic activities and these up-regulated genes. In addition, *CsePsaA-7*, the gene involved in photosynthesis, was still promoted by the TS lighting (Figure 10C), which may have acted as the up-regulator of RuBisCO activation. Furthermore, in chrysanthemums cultivated with the TS lighting, two genes associated with flowering (*Cse sc015873.1 g020.1* and *Cse sc001459.1 g030.1*) showed an extraordinary performance and followed the same pattern as that of flower induction (Figure 2A,B,E,F, and Figure 10D). Overall, those genes were significant regulators of the carbon production, photosynthesis, and flower induction in chrysanthemums, and the TS lighting increased their development.

## 4. Materials and Methods

### 4.1. Plant Growth and Treatment Design

The non-rooted cuttings of chrysanthemum (*Chrysanthemum morifolium* Ramat.) “Pearl Egg” and “Gaya Glory”, a qualitative SD plant) were obtained from the Flowers Breeding Research Institute, Gyeongnam Agricultural Research & Extension Services (GARES), Republic of Korea, in early September 2021. The cuttings were stuck in a commercial medium (BVB Medium, Bas Van Buuren Substrates, EN-12580, De Lier, The Netherlands) in 21-cell zigzag trays (21-Zigpot/21 cell tray, Daeseung, Jeonju, Korea). All cuttings were kept on a fogged propagation bench with an 80% relative humidity for 15 days and were subsequently acclimated for seven days on a greenhouse bench with an average light intensity of 350 μmol∙m^−2^∙s^−1^ PPFD of sunlight, and a natural photoperiod. The chrysanthemum plants with 8 ± 1 leaves per plant were transplanted into 10 cm plastic pots for the subsequent experiments.

**Figure 11:**
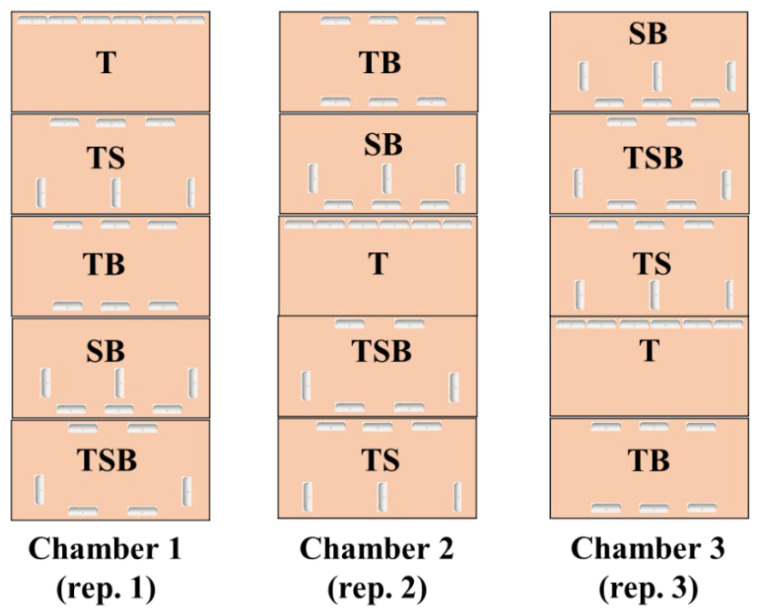
The experimental layout and design of the lighting direction combinations in plant growth chambers. T, TS, TB, SB, and TSB, refer to the top (1/1), top (1/2) + side (1/2), top (1/2) + bottom (1/2), side (1/2) + bottom (1/2), and top (1/3) + side (1/3) + bottom (1/3) lighting, respectively.

After acclimation, the transplanted seedlings were randomly divided into 15 groups (each group contained 12 plants (six plants per cultivar)) and transferred into three separate plant growth chambers (C1200H3, FC Poibe Co., Ltd., Seoul, Korea) for use as three repetitions (each chamber contained five lighting direction combinations, respectively) at 25 °C temperature with an 80% relative humidity. Each chamber was divided equally into five compartments using plates according to the lighting direction combinations (top (1/1) (T), top (1/2) + side (1/2) (TS), top (1/2) + bottom (1/2) (TB), side (1/2) + bottom (1/2) (SB), and top (1/3) + side (1/3) + bottom (1/3) (TSB), respectively). The five lighting direction combinations were randomly distributed in each chamber to avoid the effects of the position. To prevent light from interacting with each other, all light-reflecting portions inside the chambers, as well as the plates of each layer, were enclosed in an opaque black curtain. Every plate (one lighting direction combination per plate) contained one group of plants with 10 cm between each plant. The tailor-made LED lamps (SungKwang LED Co., Ltd., Incheon, Korea) with a wide spectrum ranging from 400 to 720 nm and a distinct peak at 435 nm (blue) were employed from 08:00 to 18:00 (SD condition) by adjusting the timer. Moreover, the LED lamps were fixed 10 cm from the plants in various directions. The light intensity was measured with a quantum radiation probe (FLA 623 PS, ALMEMO, Holzkirchen, Germany) at the top-leaf level of the plant [39]. In addition, the light intensity design of each lighting direction combination is shown in Figure 11 and Table 4.

The plants were watered every day at 09:00 from 23 September to 6 November 2021 with a nutrient solution composed of (in mg∙L^−1^) 708.0 Ca(NO_3_)_2_∙4H_2_O, 246.0 MgSO_4_∙7H_2_O, 505.0 KNO_3_, 230.0 NH_4_H_2_PO_4_, 1.24 H_3_BO_3_, 0.12 CuSO_4_∙5H_2_O, 4.00 Fe-ethylene diamine tetraacetic acid, 2.20 MnSO_4_∙4H_2_O, 0.08 H_2_MoO_4_, and 1.15 ZnSO_4_∙7H_2_O. Additionally, our study was not only designed as a completely randomized layout but also had 18 biological replications with consistent growth to minimize external influences.

### 4.2. Measurements of the Growth Parameters

The growth parameters were analyzed after 45 days of growth. For physiological investigations, the plants were harvested and immediately put in liquid nitrogen and kept in a −80 °C refrigerator. To analyze the growth characteristics, whole plants were collected, and the roots were thoroughly cleaned with tap water before being separated from the shoot. Except for the dry weights of the shoots and roots, the plant growth parameters shown in Table 1 were measured directly. After drying for seven days at 65 °C in a dry oven, the dry weights of the shoots and roots were measured.

### 4.3. Leaf Anatomical Features

For each lighting direction combination, six leaf segments (1 cm^2^) without midribs were collected from fully expanded leaves at the same stage in the treated plants. These segments were fixed for three days at 4 °C in a formaldehyde solution containing 5% (*v/v*) formalin, 5% (*v/v*) acetic acid, and 90% (*v/v*) ethanol. The leaf samples were dehydrated three times in a graded series of ethanol solutions (95, 75, 50, 25, and 10% (*v/v*) ethanol for each treatment for 40 min before being sliced to an appropriate thickness using the freehand slice method. The slices were mounted on glass slides and observed without staining using an optical microscope (ECLIPSE Ci-L, Nikon Corporation, Tokyo, Japan). ImageJ was used to estimate the thicknesses of the leaves, palisades, and spongy tissues.

### 4.4. Epidermal Cell and Stomatal Characteristics

Six plants were randomly selected from each lighting direction combination, and the upper and lower epidermis of leaves without midribs were carefully removed from fully expanded leaves from a similar position to observe the epidermal and stomatal morphology. The stomata were observed by tearing the epidermis off the leaf with gummed tape [73]. The excised samples were observed with an optical microscope (ECLIPSE Ci-L, Nikon Corporation, Tokyo, Japan) at different magnifications and analyzed with ImageJ. The stomatal density was calculated by dividing the number of stomata by the area where the number of stomata was recorded. The length and width of guard cell pairs and stomatal pores were measured according to the definition in Sack and Buckley [19].

### 4.5. Photosynthesis and Chlorophyll Contents

The plants were kept in the plant growth chambers in which the net photosynthetic rate (*P*_n_), transpiration rate (*T*_r_), stomatal conductance (*G*_s_), and intercellular CO_2_ concentration (*C*_i_) were measured on the youngest mature leaf of each plant with a leaf porometer (SC-1, Decagon Device Inc., Pullman, WA, USA). For this purpose, a 3 cm^2^ area of a terminal leaflet was enclosed in a leaf chamber mounted horizontally. The lighting intensity in the plant growth chambers was 600 μmol∙m^−2^·s^−1^ (PPFD), relative humidity about 80%, and the CO_2_ concentration was the same as outside.

0.1 g fresh leaf samples were collected for the chlorophyll content measurements, and six replicates were used for each lighting direction combination. All samples were dipped in 10 mL of N,N-dimethyl formamide solution in the dark for 48 h at 4 °C, and then Chl a and Chl b contents were measured. The absorbances of the upper layer solution at 645 and 663 nm were recorded with a UV spectrophotometer (Libra S22, Biochrom Ltd., Cambridge, UK). The chlorophyll content was calculated in accordance with the method of Sim et al. [74]. Moreover, the chlorophyll content was expressed as the chlorophyll/fresh leaf weight (mg/g).

### 4.6. Chlorophyll Fluorescence Measurements

The miniaturized pulse-amplitude-modulated photosynthesis yield was used to detect the chlorophyll fluorescence. Each plant was moved to a dark chamber for 30 min to adapt before being measured with a photosystem (Fluor Pen FP 100, Photon Systems Instruments, PSI, Drásov, Czech Republic). The measurements involved the maximal PSII quantum yield (*F*_v_/*F*_m_), photochemical efficiency of PSII (*F*_v_′/*F*_m_′), non-photochemical quenching (NPQ), and coefficient of photochemical quenching (*qP*). All the parameters were calculated using the methods reported by Maxwell et al. [75].

### 4.7. Contents of Carbohydrates and Soluble Proteins

For carbohydrate measurements, leaves at the same stage were taken at the end of the day or night. The Anthrone colorimetric method according to Vasseur and Ren et al. was used for the starch and soluble sugar measurements [76,77]. The method for extracting soluble proteins is as described: Fresh leaves were collected, immediately immersed in liquid nitrogen, and ground into a fine powder over an ice bath. 100 mg of the powder was homogenized in 50 mM of PBS (1 mM EDTA, 1 mM polyvinylpyrrolidone, and 0.05% (*v/v*) triton-X, pH = 7.0). The resulting mixture was then centrifuged (13,000 rpm, 4 °C, 20 min) to obtain the supernatant that would be used afterward for the total protein estimation and enzyme activity assay [78]. The total protein estimations were conducted using Bradford’s reagent [79,80]. The contents of carbohydrates and soluble proteins were measured with a UV spectrophotometer (Libra S22, Biochrom Ltd., Cambridge, UK).

### 4.8. Enzyme Activities

Superoxide dismutase activity (SOD) was assayed as described by Becana et al. [81], monitoring its ability to inhibit the photochemical reduction of nitroblue trtrazolium (NBT). The 3 mL reaction mixture solution contained a 50 mM potassium phosphate buffer (PH 7.8), 50 mM methionine, 75 μM NBT, 20 μM riboflavin, 0.1 mM EDTA, and 0.1 mL of the enzyme extract. The reaction mixture assay was performed at 120 μmol m^−2^ s^−1^ for 15 min. Blanks and the control were run similarly, but without illumination and the enzyme extract, respectively. One unit of SOD was defined as the amount of enzyme which produced a 50% inhibition of NBT reduction by monitoring at A_560 nm_.

Catalase (CAT) activity was assayed according to Aeibi et al. [82]. The induction of H_2_O_2_ in A_240 nm_ was directly measured in the homogenates, and the reaction medium contained a 50 mM potassium phosphate buffer (PH 7.0), 10 mM H_2_O_2_, and 0.1 mL enzyme extract in a final volume of 3 mL at 25 °C. The CAT activity was determined using the extinction coefficient (40 mM^−1^ cm^−1^) for H_2_O_2_.

Ascorbate peroxidase activity (APX) was analyzed according to Nakano [83] by monitoring the decrease at A_290 nm_ (extinction coefficient 2.9 mM^−1^ cm^−1^) for 1 min. The reaction mixture contained a 50 mM sodium phosphate buffer (PH 7.0), 0.1 mM EDTA, 1 mM ascorbate acid, 2.5 mM H_2_O_2_, and 50 μL of the enzyme extract.

Peroxidase activity (POD) was measured based upon the method described by Castillo et al. [84]. The 3 mL reaction mixture contained 10 mM guaiacol, 50 mM H_2_O_2_, a 50 mM phosphate buffer (PH 6.0), and 50 μL enzyme extract. The absorbance was recorded at A_470 nm_ and was calculated using the extinction coefficient 26.6 mM^−1^ cm^−1^.

The enzymatic activities of the key enzymes related to sucrose synthesis (SS and PEPC), starch synthesis (SSS), and photosynthesis (RuBisCO) were measured using a UV spectrophotometer (Libra S22, Biochrom Ltd., Cambridge, UK). The SS was determined in a 1 mL reaction mixture containing 500 μL enzyme extract at 34 °C for 1 h. A 300 μL 30% (*v/v*) KOH was added to this mixture, followed by putting it in a water bath at 100 °C for 10 min and gradually cooling to room temperature. The mixture was subjected to incubation at 40 °C for 20 min after 200 μL 0.15% (*v/v*) anthrone-sulfuric acid solution was applied and the enhancement of A_620 nm_ was monitored. The total RuBisCO activity was measured by injecting 100 μL of the supernatant into 400 μL of an assay mixture consisting of 50 mM Tris-HCl (pH 8.0), 5 mM DTT, 10 mM MgCl_2_, 0.1 mM EDTA, and 20 mM NaH_14_CO_3_ (2.0 GBq mmol^−1^) at 30 °C. After a 5-min activation period, the reaction was initiated by adding RuBP to 0.5 mmol L^−1^ and terminated after 30 s with 100 μL of 6 mol L^−1^ HCl. The PEPC was assayed in a 1 mL reaction mixture consisting of 50 mM Tris-HCl (pH 8.0), 5 mM MnCl_2_, 2 mM DTT, 10 mM NaHCO_3_, 0.2 mM NADH, 5 units of NAD-MDH, and 160 μL of the enzyme extract. The reaction was initiated by adding 2.5 mM phosphoenolpyruvate (PEP), and the increase in the A_412 nm_ was monitored. The above description of enzymatic activities was as described in Feng et al. and Yang et al. [85,86]. In addition, activities of the soluble starch synthase (SSS) were measured according to the protocol described by Doehlert et al. and Liang et al. [87,88].

### 4.9. Real-Time Quantitative PCR Verification

The expression of key genes related to sucrose synthesis (*CseSSS-4)*, starch synthesis (*CseSS-7*), photosynthesis (*CsePsaA-7*), and flowering (*Cse_sc015873.1_g020.1* and *Cse_sc001459.1_g030.1*) were chosen from “https://plantgaden.jp/en/list/t1111766” and http://mum-garden.kazusa.or.jp/ (accessed on 27 November 2021). All the leaves were immediately frozen in liquid nitrogen. The total RNA was extracted using an Easy-Spin total RNA extraction kit (iNtRON Biotechnology, Seoul, Korea), then used for first-stand cDNA synthesis with the GoScript Reverse Transcription System (Promega, Madison, WI, USA) according to the manufacturer’s protocols. Real-time quantitative PCR was conducted in a real-time PCR system (CFX96, Bio-Rad, Hercules, CA, USA). Reaction volumes (20 μL) contained 1 μL of cDNA, 1 μL of each amplification primer (10 μM), 10 μL of 2 × AMPIGENE qPCR Green Mix Lo-ROX (Enzo Life Sciences Inc., Farmingdale, NY, USA), and 7 μL ddH_2_O (double distilled water). The 2−ΔΔCt method was used for the data analysis, and the *ACTIN* gene (*Cse_sc001321.1_g010.1*) was selected as the control. All the target gene primers are listed in Table 5.

### 4.10. Statistical Analysis

Significant differences among the treatments were assessed by an analysis of variance (ANOVA) followed by Duncan’s multiple range test at a probability (*p*) < 0.05 with a statistical program (SAS, Statistical Analysis System, V. 9.1, Cary, NC, USA). The Fisher’s least significant difference test was used for the *F*-test between the treatments. The experimental assays used to obtain all results were repeated six times and are presented as the mean ± standard error.

## 5. Conclusions

The significant effects of the lighting quality, quantity, and intensity on plants have been extensively investigated, but researchers have rarely studied the impacts of different lighting directions on chrysanthemum to understand the optimal combination of various lighting directions for better growth and development. In our study, differences in the lighting direction combinations induced epinastic or hyponastic leaf movements in chrysanthemums to efficiently capture the available light, resulting in variations in the leaf petiole angle, which is the threshold point that strongly regulates the morphophysiology. The TS lighting significantly improved the morphological characteristics, carbohydrate assimilation rate, key enzymatic activities, and other plant performances by up-regulating the correlated genes. Moreover, the excellent early flower induction occurred in response to the TS lighting by up-regulating the flowering-related genes (*Cse sc015873.1 g020.1* and *Cse sc001459.1 g030.1*) in both “Pearl Egg” and “Gaya Glory” plants. Particularly, when compared to the SB lighting, the TS lighting significantly enhanced leaf development, which helps improve the chlorophyll fluorescence and quantum yield of PSII, which in turn substantially promoted photosynthesis and increased chrysanthemum growth and development. Altogether, the TS lighting more properly changed the leaf orientation and adjusted the leaf angle to capture as much of the available light as possible, inducing better plant performances, especially splendid flowering and branching. Thus, the TS lighting provided the optimal lighting conditions in our experiment for chrysanthemums and obviously improved the light using efficiency. However, further investigation remains necessary to explore the involved internal signaling pathways (e.g., hormone signaling).

## Figures and Tables

**Table 1:** The influence of the lighting direction combinations on the growth and development of chrysanthemums grown for 45 days.

Cultivar (A)	Treatment (B)	Shoot								
		Plant Height(cm)	Canopy Diameter (cm)	No. of Branches		Stem Diameter (mm)	No. of Nodes	Length of Top 5th Internode (mm)	Fresh Weight (g)	Dry Weight (g)
Pearl Egg	T ^1^	12.23 c ^2^	12.44 d	8.33 e		2.72 c	15.00 d	5.06 d	18.46 e	1.84 e
	TS	14.73 a	9.19 g	11.33 d		3.09 ab	23.00 b	5.68 bc	24.95 d	2.50 d
	TB	13.00 b	8.22 h	5.67 f		2.73 c	11.67 e	5.79 b	15.11 f	1.51 f
	SB	10.60 e	4.22 j	2.67 g		2.28 d	15.33 d	4.93 e	8.21 h	0.74 h
	TSB	10.17 f	9.83 f	8.67 e		2.70 c	19.00 c	4.25 f	15.16 f	1.52 f
Gaya Glory	T	10.90 d	12.87 c	16.00 c		3.06 b	16.00 d	5.64 c	31.90 c	3.19 c
	TS	12.23 c	14.90 a	20.00 a		3.13 a	22.67 b	5.10 d	47.88 a	4.79 a
	TB	10.30 f	10.87 e	12.33 d		2.17 e	12.67 e	6.26 a	25.33 d	2.53 d
	SB	8.30 g	6.40 i	6.33 f		2.13 e	19.33 c	4.13 f	12.94 g	1.29 g
	TSB	12.27 c	14.20 b	17.67 b		2.75 c	27.00 a	4.00 g	40.75 b	4.13 b
*F*-test	A	***	***	***		***	***	***	***	***
	B	***	***	***		***	***	***	***	***
	A × B	***	***	***		***	***	***	***	***
**Cultivar (A)**	**Treatment (B)**	**Leaf**				**Flower**		**Root**		
		**No. of** **Leaves**	**Adaxial** **Petiole Angle** **(°)**	**Length** **(cm)**	**Width** **(cm)**	**No. of Flowers**	**DVB ^3^** **(Day)**	**Length** **(cm)**	**Fresh Weight (g)**	**Dry Weight** **(g)**
Pearl Egg	T ^1^	93.33 ef ^2^	60.63 f	3.78 c	2.49 de	18.00 ef	16.67 h	31.37 d	2.81 c	0.29 c
	TS	105.33 d	75.90 e	4.77 a	3.09 a	23.67 d	13.33 i	38.87 a	3.61 a	0.36 a
	TB	83.67 g	95.33 cd	3.38 g	2.19 g	19.00 ef	20.33 g	27.31 e	2.14 e	0.21 e
	SB	59.67 i	121.27 a	3.31 h	2.04 i	7.33 g	29.67 c	17.91 g	1.10 g	0.11 f
	TSB	88.67 fg	71.57 e	3.71 d	2.46 e	17.00 f	20.00 g	27.41 e	2.14 e	0.21 e
Gaya Glory	T	121.00 c	41.83 g	3.60 e	2.60 c	31.33 c	23.67 e	32.37 c	2.71 d	0.28 d
	TS	171.67 a	90.23 d	4.08 b	2.85 b	82.33 a	21.33 f	33.87 b	3.51 b	0.35 b
	TB	97.00 e	100.17 c	3.11 i	2.39 f	20.67 de	26.33 d	25.31 f	2.04 f	0.20 e
	SB	74.67 h	111.53 b	2.89 j	2.14 h	8.00 g	37.67 a	15.57 h	1.01 h	0.10 f
	TSB	164.67 b	70.43 e	3.43 f	2.50 d	55.33 b	32.67 b	25.41 f	2.07 f	0.21 e
*F*-test	A	***	NS	***	***	***	***	***	***	***
	B	***	***	***	***	***	***	***	***	***
	A × B	***	***	***	***	*	***	**	NS	NS

^1^ T, TS, TB, SB, and TSB, refer to the top (1/1), top (1/2) + side (1/2), top (1/2) + bottom (1/2), side (1/2) + bottom (1/2), and top (1/3) + side (1/3) + bottom (1/3) lighting, respectively. ^2^ Mean separation within columns by Duncan’s multiple range test at *p* ≤ 0.05. ^3^ Days of treatment to the visible flower buds. NS, *, **, ***, non-significant or significant at *p* ≤ 0.05, 0.01, or 0.001, respectively.

**Table 2:** Influence of the lighting direction combinations on the photosynthetic characteristics of chrysanthemums grown for 45 days.

Cultivar (A)	Treatment (B)	*P*_n_^3^ (μmol CO_2_ m^−2^·s^−1^)	*T*_r_^4^ (mmol H_2_O m^−2^·s^−1^)	*G*_s_^5^ (mol H_2_O m^−2^·s^−1^)	*C*_i_^6^ (μmol CO_2_ mol^−1^)
Pearl Egg	T ^1^	19.00 d ^2^	2.10 d	0.74 d	449.73 d
	TS	19.73 c	2.14 c	0.78 c	456.57 c
	TB	17.53 e	2.08 e	0.72 e	435.10 e
	SB	13.03 g	1.39 g	0.37 f	393.50 g
	TSB	17.57 e	2.08 e	0.71 e	435.10 e
Gaya Glory	T	19.87 bc	2.16 c	0.79 bc	458.60 c
	TS	21.93 a	2.22 a	0.86 a	490.17 a
	TB	19.63 c	2.15 c	0.77 c	456.10 c
	SB	16.30 f	1.92 f	0.72 de	401.90 f
	TSB	20.13 b	2.19 b	0.81 b	467.93 b
*F*-test	A	***	***	***	***
	B	***	***	***	***
	A × B	***	***	***	***

^1^ T, TS, TB, SB, and TSB, refer to the top (1/1), top (1/2) + side (1/2), top (1/2) + bottom (1/2), side (1/2) + bottom (1/2), and top (1/3) + side (1/3) + bottom (1/3) lighting, respectively. ^2^ Mean separation within columns by Duncan’s multiple range test at *p* ≤ 0.05. ^3^ Net photosynthetic rate. ^4^ Transpiration rate. ^5^ Stomatal conductance. ^6^ Intercellular CO_2_ concentration. ***, significant at *p* ≤ 0.001.

**Table 3:** Influence of the lighting direction combination on the chlorophyll fluorescence characteristics of chrysanthemums grown for 45 days.

Cultivar (A)	Treatment (B)	*F*_v_/*F*_m_^3^	*F*_v_′/*F*_m_′ ^4^	NPQ ^5^	*qP* ^6^
Pearl Egg	T ^1^	0.82 e ^2^	0.66 d	2.76 c	0.54 c
	TS	0.87 d	0.71 c	2.79 c	0.59 b
	TB	0.78 f	0.52 e	2.47 d	0.45 d
	SB	0.60 h	0.41 g	1.98 f	0.33 f
	TSB	0.79 f	0.53 e	2.50 d	0.45 d
Gaya Glory	T	0.90 c	0.72 c	2.81 c	0.58 b
	TS	0.99 a	0.79 a	2.97 a	0.68 a
	TB	0.86 d	0.70 c	2.78 c	0.57 b
	SB	0.68 g	0.46 f	2.07 e	0.39 e
	TSB	0.93 b	0.75 b	2.89 b	0.66 a
*F*-test	A	***	***	***	***
	B	***	***	***	***
	A × B	***	***	***	***

^1^ T, TS, TB, SB, and TSB, refer to the top (1/1), top (1/2) + side (1/2), top (1/2) + bottom (1/2), side (1/2) + bottom (1/2), and top (1/3) + side (1/3) + bottom (1/3) lighting, respectively. ^2^ Mean separation within columns by Duncan’s multiple range test at *p* ≤ 0.05. ^3^ The maximal PSII quantum yield (*F*_v_/*F*_m_). ^4^ The photochemical efficiency of PSII (*F*_v_′/*F*_m_′). ^5^ Non-photochemical quenching (NPQ). ^6^ Coefficient of photochemical quenching (*qP*). ***, significant at *p* ≤ 0.001.

**Table 4:** Light intensity design of the lighting direction combinations.

Lighting Direction	Abbreviation	Light Intensity (μmol∙m^−2^∙s^−1^ Photosynthetic Photon Flux Density (PPFD))
Top (1/1)	T	600
Top (1/2) + Side (1/2)	TS	300 per lighting direction
Top (1/2) + Bottom (1/2)	TB	300 per lighting direction
Side (1/2) + Bottom (1/2)	SB	300 per lighting direction
Top (1/3) + Side (1/3) + Bottom (1/3)	TSB	200 per lighting direction

**Table 5:** List of the primers used to quantify the gene expression levels.

Name	Gene ID	GO ID	Forward Primer (5′ to 3′)	Reverse Primer (5′ to 3′)
*ACTTIN* *Cse_sc001321.1_g010.1*	3641_0:00292c	GO:0005524	AACTGGGACGATATGGAGAAGA	CGCAAGATAGCATGTGGAAGTG
*SS-7* *Cse_sc012707.1_g010.1*	4232_0:0027ec	GO:0009507	GGCCTTGGAGCAAAACTGGT	AGTCTATTCCAGCAACAGGTCC
*SSS-4* *Cse_sc005354.1_g010.1*	4232_0:003c28	GO:0016157	TGAGAATATGTGCTGGCGGA	TCGCACCAACCCATGGATAC
*PsaA-7* *Cse_sc003237.1_g010.1*	4232_0:00aa9f	GO:0016021	ACTGGTAGTGGTGGGAAAGC	CTTAGAGCCTGAGCATCTGAGT
*Cse_sc015873.1_g020.1*	4236_0:004cfa	GO:0009909	ATGTCTGGTGTTTGGGTGTTTA	CTACATATCTTACTTCAA
*Cse_sc001459.1_g030.1*	4232_0:00932a	GO:0042753	GATGGCAAGGTGATGCAAACA	TCGAAAAATTCGACGAAAGATCC

## Data Availability

Data sharing is not applicable to this article.
